# U6 snRNA Pseudogenes: Markers of Retrotransposition Dynamics in Mammals

**DOI:** 10.1093/molbev/msv062

**Published:** 2015-03-11

**Authors:** Aurélien J. Doucet, Gaëtan Droc, Oliver Siol, Jérôme Audoux, Nicolas Gilbert

**Affiliations:** ^1^Institut de Génétique Humaine, CNRS, UPR 1142, Montpellier, France; ^2^Institute for Research on Cancer and Aging, Nice (IRCAN), INSERM, U1081, CNRS UMR 7284, Nice, France; ^3^Centre de Coopération Internationale en Recherche Agronomique pour le Développement (Cirad), UMR AGAP, Montpellier, France; ^4^Institute for Regenerative Medicine and Biotherapy, INSERM, U1183, Montpellier, France

**Keywords:** retrotransposon, long interspersed nuclear element, small nuclear RNA

## Abstract

Transposable elements comprise more than 45% of the human genome and long interspersed nuclear element 1 (LINE-1 or L1) is the only autonomous mobile element remaining active. Since its identification, it has been proposed that L1 contributes to the mobilization and amplification of other cellular RNAs and more recently, experimental demonstrations of this function has been described for many transcripts such as *Alu*, a nonautonomous mobile element, cellular mRNAs, or small noncoding RNAs. Detailed examination of the mobilization of various cellular RNAs revealed distinct pathways by which they could be recruited during retrotransposition; template choice or template switching. Here, by analyzing genomic structures and retrotransposition signatures associated with small nuclear RNA (snRNA) sequences, we identified distinct recruiting steps during the L1 retrotransposition cycle for the formation of snRNA-processed pseudogenes. Interestingly, some of the identified recruiting steps take place in the nucleus. Moreover, after comparison to other vertebrate genomes, we established that snRNA amplification by template switching is common to many LINE families from several LINE clades. Finally, we suggest that U6 snRNA copies can serve as markers of L1 retrotransposition dynamics in mammalian genomes.

## Introduction

Mobile elements, known as transposons and retrotransposons, make up a large fraction of all eukaryotic genomes. Non-LTR (long terminal repeat) retrotransposons are present in most eukaryotes and are divided into 28 clades based on phylogenetic analysis (Malik et al. 1999; Eickbush and Malik 2002; Kapitonov et al. 2009). In vertebrates, the four major clades are L1, L2, CR1, and RTE. For over 100 My, long interspersed nuclear elements 1 (known as LINE-1 or L1), from the L1 clade, have sculpted *Metatheria* and *Eutheria* genomes, representing between 15% and 20% of the DNA, while being almost absent in *Prototheria* genomes (Smit 1996; Lander et al. 2001; Lindblad-Toh et al. 2005; Mandal and Kazazian 2008; Warren et al. 2008). In the human genome, L1 is believed to be the only autonomous mobile element remaining active, and it continues to have a mutagenic impact by various mechanisms including insertion, duplication, deletion, and recombination (Deininger et al. 2003; Chen et al. 2005; Babushok and Kazazian 2007; Jurka et al. 2007; Muotri et al. 2007; Cordaux and Batzer 2009; Xing et al. 2009; Beck et al. 2010; Ewing and Kazazian 2010; Huang et al. 2010; Iskow et al. 2010; O’Donnell and Burns 2010; Baillie et al. 2011). Although never observed, human endogenous retrovirus-K (HERV-K), an LTR retrotransposon, may theoretically be active as functional copies have the potential to exist in individual genomes (Dewannieux et al. 2006; Ruprecht et al. 2008; Hohn et al. 2013).

It is estimated that the average human genome contains approximately 80–100 Retrotransposition Competent L1s (RC-L1) (Brouha et al. 2003; Beck et al. 2010; Macfarlane et al. 2013). A human RC-L1 produces a 6-kb transcript from an internal promoter, with two nonoverlapping open reading frames (ORF) (Scott et al. 1987; Swergold 1990; Dombroski et al. 1991; Athanikar et al. 2004; Lavie et al. 2004). The two proteins produced from the L1 RNA, ORF1p and ORF2p, are essential for L1 retrotransposition (Moran et al. 1996). ORF1p contains a coiled-coil domain required for its multimerization and an RNA binding domain involved in the formation of a ribonucleoprotein particle (RNP) complex with the L1 RNA and ORF2p (Hohjoh and Singer 1996; Martin et al. 2005; Kulpa and Moran 2006; Khazina and Weichenrieder 2009; Doucet et al. 2010). ORF2p contains the endonuclease (EN) and reverse transcriptase (RT) domains required for autonomous retrotransposition (Mathias et al. 1991; Feng et al. 1996; Cost et al. 2002). Both proteins essentially act in *cis* to form an RNP complex with their encoding L1 RNA (Esnault et al. 2000; Wei et al. 2001; Kulpa and Moran 2005, 2006; Doucet et al. 2010; Goodier et al. 2010). The RNP enters the nucleus and then mediates a new L1 insertion through a mechanism known as target-site primed reverse transcription (TPRT) (Luan et al. 1993; Feng et al. 1996; Cost and Boeke 1998; Cost et al. 2002; Christensen and Eickbush 2005; Kulpa and Moran 2006). Briefly, the EN domain of ORF2p cleaves the DNA and the RT domain concomitantly produces L1 cDNA using L1 RNA as a template and the genomic DNA cleavage as a primer. Hallmarks of the process are the consensus cleavage site (5′-TTTT/A), a variable length of L1 poly(A), and the presence of target site duplication (TSD) on both sides of the often 5′-truncated new L1 copy (Gilbert et al. 2002).

The human genome contains numerous copies of pseudogenes from coding or noncoding genes, and it was proposed that a majority of them have been generated through the processing of an RNA intermediate (Denison et al. 1981; Van Arsdell et al. 1981; Bernstein et al. 1983; Vanin 1985). More recently, it has been demonstrated that most of the pseudogenes, defined by 1) the absence of intronic sequences and 2) the presence of scattered base mismatches compared with the corresponding parental coding gene sequence, were amplified through L1-mediated reverse transcription. Indeed, even if RC-L1 proteins show a strong *cis*-preference to mobilize their encoding RNA, they can act in *trans* to amplify nonautonomous retrotransposons (i.e., short interspersed nuclear elements or SINEs), cellular mRNAs, and small noncoding RNAs such as tRNAs and uracil-rich small nuclear RNAs (i.e., small nuclear [sn], small nucleolar [sno], and Y RNAs) (Rogers 1985; Maestre et al. 1995; Esnault et al. 2000; Wei et al. 2001; Buzdin et al. 2002, 2003; Dewannieux et al. 2003; Zhang et al. 2003; Schmitz et al. 2004; Gilbert et al. 2005; Gogvadze et al. 2005; Perreault et al. 2005; Weber 2006; Garcia-Perez et al. 2007). In general, the formation of processed pseudogenes requires the expression of both L1 proteins (Esnault et al. 2000; Wei et al. 2001; Garcia-Perez et al. 2007). In contrast, ORF1p is dispensable for the amplification of the SINE *Alu*, the most abundant nonautonomous retroelement of our genome (Dewannieux et al. 2003). Although, another study suggests that ORF1p may enhance *Alu* mobilization (Wallace et al. 2008).

In light of these data, distinct mechanisms have been proposed to explain the *trans*-mediated mobilization of cellular RNAs by the L1 machinery (Sinnett et al. 1992; Boeke 1997; Buzdin et al. 2002; Dewannieux et al. 2003; Schmitz et al. 2004). A closer analysis of 3′-flanking sequences of small noncoding RNA pseudogenes and the detection of L1 retrotransposition signatures revealed that at least two L1-dependent mechanisms could be involved in *trans*-mobilization events (Buzdin et al. 2002, 2003; Schmitz et al. 2004; Gilbert et al. 2005; Perreault et al. 2005; Garcia-Perez et al. 2007; Lucier et al. 2007). The main mechanism involves mobilization by template choice, that is, L1 proteins bind and initiate reverse transcription directly on the mobilized RNA (Schmitz et al. 2004; Perreault et al. 2005; Garcia-Perez et al. 2007). A second mechanism involves mobilization by template switching, that is*,* the reverse transcription is initiated at the L1 RNA poly(A) tail and is later followed by a substitution of the RNA template used to generate cDNA. This second mechanism seems to be restricted to a limited number of snRNAs (Buzdin et al. 2002, 2003; Gilbert et al. 2005; Garcia-Perez et al. 2007). Processed pseudogenes formed by template switching are called chimeras.

Here, we retrieved pseudogenes of snRNA genes that are part of spliceosomal complexes by screening mammalian genomes with available sequencing data. These snRNAs are short sequences (between 100 and 200 bases) and are highly conserved among vertebrates (see Materials and Methods). After transcription they are subjected to modifications, and once maturated, they are involved in RNP complexes that are requested for excising introns from cellular mRNA (Patel and Steitz 2003; Kiss 2004; Matera and Wang 2014). We were able to classify the snRNA pseudogenes in groups depending on the distinct signature pattern of each sequence. We observed that the vast majority of pseudogenes was amplified through an L1-dependent mechanism. We further established that the distinction between groups of processed pseudogenes most likely reflects differences in RNA recruitment during the process of L1-mediated retrotransposition. Here, we propose new mechanisms by which L1 can mobilize cellular RNAs that have subsequently contributed to the architecture of mammalian genomes. Furthermore, even though retrotransposition pathways are conserved among placental mammalian genomes, we were able to highlight the variability of retrotransposition dynamics among mammalian species. We further propose the use of U6 snRNA sequence as a marker of L1 activity. Finally, by analyzing other vertebrate genomes, we established that the template switching mechanism to amplify U6 snRNA is not restricted to LINEs from the evolutionary conserved L1 clade.

## Results and Discussion

### snRNA Genomic Copies

To understand the mechanisms that mediate the mobilization of cellular RNAs to form processed pseudogenes, we characterized insertion sites of snRNA sequences in the human genome. Following a previous study (Garcia-Perez et al. 2007), we selected gene sequences of the nine snRNAs involved in major and minor splicing complexes (i.e., U1, U2, U4, U5, U6, U11, U12, U4atac, and U6atac). For each, we conducted BLAST (Basic Local Alignment Search Tool) searches of the human genome working draft sequence (see Materials and Methods). In order to restrict false positive hits and to limit the number of sequences to analyze, we filtered the search to only keep sequences that present less than 10% divergence from the reference gene (3,512 sequences). Due to a much larger number of hits obtained for U2 and U6, we further limited our analysis to sequences with at least 97.5% identity. We next applied selective parameters (see Materials and Methods) to sort 450 sequences out of the 3,512 copies originally retrieved. The sorted sequences were fully characterized (table 1). From these, 256 sequences corresponded to our selective criteria and almost 80% of them presented variable-sized TSD flanking the integrated sequence, strongly suggesting the implication of L1 retrotransposons in the formation of these processed pseudogenes.

**Table 1.** Distribution of snRNA Copies in the Human Genome.

	Hits^a^	Analyzed^b^	Characterized^c^	Alone	Repeat	Poly(A)	3′-trunc
U1	88	53	33	4 (12.1)	1 (3.0)	18 (54.5)	10 (30.3)
		With TSD	20 (60.6)		1 (100)	12 (66.7)	7 (70.0)
U2*	1,708	143	58		1 (1.7)		57 (98.3)
		With TSD	51 (87.9)		1 (100)		50 (87.7)
U4	305	64	32	2 (6.3)		9 (28.1)	21 (65.6)
		With TSD	25 (78.1)			6 (66.7)	19 (90.5)
U5	360	94	67	3 (4.5)	7 (10.4)	1 (1.5)	56 (83.6)
		With TSD	57 (85.1)		7 (100)	1 (100)	49 (87.5)
U6*	906	55	36	4 (11.1)	14 (38.9)	6 (16.7)	12 (33.3)
		With TSD	31 (86.1)		13 (92.9)	6 (100)	12 (100)
U4atac	55	6	3	2			1
U6atac	63	29	23	2 (8.7)	7 (30.4)	6 (26.1)	8 (34.8)
		With TSD	16 (69.6)		6 (85.7)	3 (50)	7 (87.5)
U11	12	2	2	1			1
U12	15	4	3	2			1
Total	3,512	450	256	20 (7.8)	30 (11.7)	40 (15.6)	166 (64.8)
		With TSD	201 (78.5)		28 (93.3)	28 (70.0)	145 (87.3)

Note.—The names of the snRNA sequences used for BLAST search are indicated on the left. For U2 snRNA, the precursor gene is assigned to chromosome 17 but not annotated to a particular locus, thus was not included in this table. Numbers in parenthesis give the proportion in % of each type of structure per snRNA sequence. “With TSD” gives the number of sequences with identified TSD, and numbers associated in parenthesis give the proportion in % of sequences with identified TSD for each type of structure.

^a^Number of retrieved sequences with 90% identity to the reference gene.

^b^Number of analyzed sequences after applying selective parameters (see Materials and Methods section).

^c^Effective number of unique sequences characterized. The next columns give the distribution of copies depending on the identified associated sequences (Alone, sequences not associated with repeats; Repeat, sequences associated with retrotransposon; Poly(A), sequences with an A-rich 3′-extremity; 3′-trunc, 3′-truncated copies).

We observed that processed pseudogenes derived from small RNAs involved in the major splicing complex (i.e.*,* U1, U2, U4, U5, and U6) are more represented than those specific to the minor splicing complex (i.e.*,* U4atac, U6atac, U11, and U12). The latest ones correspond to 12% of the analyzed sequences (table 1). This could simply reflect the differential abundance of these transcripts in cells (Patel and Steitz 2003), and thus their potential to be recruited by L1 machinery to form processed pseudogenes. Interestingly, it has been reported that abundant ubiquitously expressed transcripts (e.g., ribosomal protein genes, cyclophilin-A, keratin, GAPDH, and cytochrome *C*) account for a large fraction of the processed pseudogenes identified in the human genome (Zhang et al. 2003).

To gain insight into the recruitment of snRNAs by the L1 machinery, we next carefully looked at the structure of the insertion site of the 256 sequences identified above. We classified each snRNA genomic copy by its flanking genomic sequence (table 1). Full-length copies not associated with any repeat sequence are in the first group (Alone). The second group comprises copies associated with retrotransposon sequences (i.e.*,* LINEs, SINEs, or processed pseudogenes) (Repeat). The third group represents snRNA sequences with an A-rich 3′-extremity (Poly(A)). Finally, the fourth set regroups 3′-truncated copies (3′-trunc). The sum of all snRNA copies for each group is represented at the bottom of table 1 (Total). We then analyzed the presence of TSD in each group. For the first group (Alone), none of the copies was found with TSD. They most likely represent active genes, when they present 100% identity to the reference gene, or genomic duplications. For example, we were able to associate the four U6 full-length copies to previously identified transcriptionally active sequences (Domitrovich and Kunkel 2003). Thus, they were not considered as the result of retrotransposition events. In contrast, most of the sequences from the other three groups are associated with detectable TSD (93%, 70%, and 87%, respectively; table 1). Based on the sequence of these TSDs, the vast majority of the cleavage sites resembles the L1 consensus cleavage site 5′-TTTT/A (data not shown). Thus, these copies are most likely derived from L1-dependent mobilization mechanisms. Moreover, by opposition of the sequences from the first group, most if not all of these retrotransposed snRNA copies become nonfunctional upon insertion as they have lost the *cis*-acting sequences required for bona fide transcription and/or maturation (Matera and Wang 2014).

### snRNA Associated with Retrotransposons

**Fig. 1.**
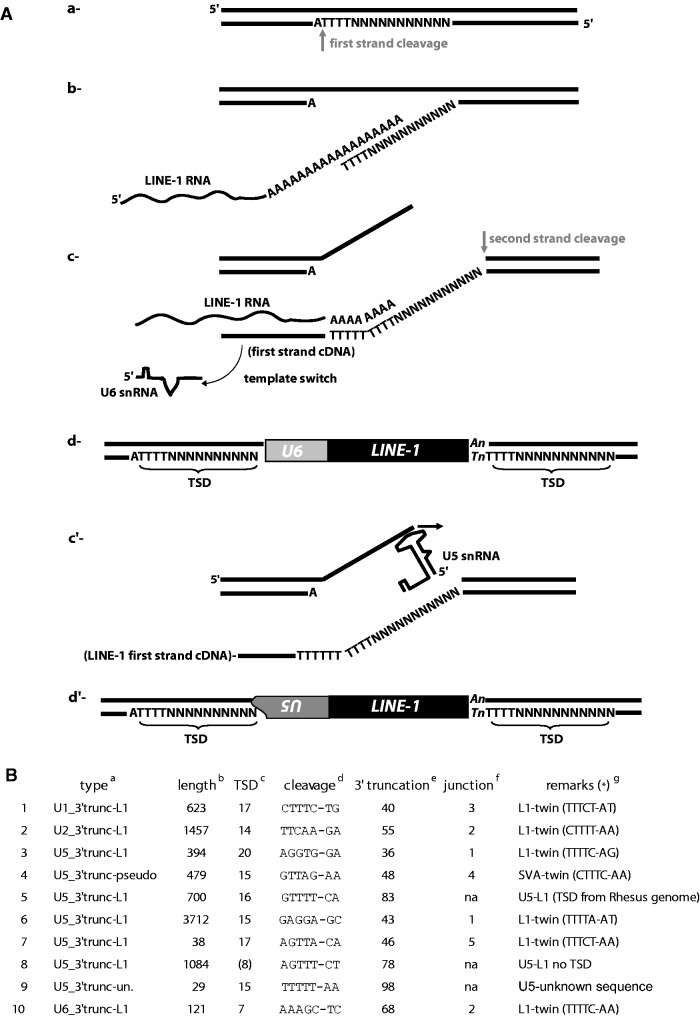
Template switch and twin priming. (*A*) Steps describing template switching (*a–d*) and twin priming (*a*, *b*, *c*′, and *d*′) mechanisms: (*a*) First strand cleavage by the L1 EN domain of ORF2p, (*b*) annealing of the L1 RNA to the cleaved site and initiation of reverse transcription, (*c*) template switching during reverse transcription from the L1 RNA to a U6 snRNA, and second strand cleavage, (*d*) resolution of the insertion that generates a chimera with a U6 copy followed by a 5′-truncated L1 sequence and flanked by TSD, (*c*′) after second strand cleavage, on the DNA top strand, initiation of reverse transcription directly on the U5 snRNA, and (*d*′) resolution of the insertion that generates a chimera with a 3′-truncated inverted U5 sequence followed by a 5′-truncated L1 sequence flanked by TSD. Note that the two sequences are in opposite transcriptional orientation. (*B*) List of the chimeras found with U1, U2, U5, and U6 snRNA that follow the twin-priming model, enumerated in column 1. ^a^snRNA type. ^b^Length of L1 sequence. ^c^Size of the TSD. ^d^Cleavage site based on the snRNA transcriptional orientation. ^e^Nucleotide number in the snRNA truncation. ^f^Number of nucleotides common to the snRNA and to the insertion site at the 5′-junction of the insertion. ^g^Chimeras type (template switching or twin priming, mentioned by “twin”). *In parenthesis, cleavage site based on the L1 transcriptional orientation. For sequence # 5, we were able to build the TSD in the human genome based on the empty site of the orthologous loci in the rhesus genome. “Unknown sequence” means that the sequence found associated with the U5 copy is not a repeated sequence as it has only been found once in the genome.

Genomic snRNA copies associated with retrotransposed sequences such as L1 or processed pseudogenes are called U/L1 or U/pseudogene chimeras (Buzdin et al. 2003; Garcia-Perez et al. 2007; Hasnaoui et al. 2009). They are the results of template switching events (fig. 1*A*[a–d]). As mentioned in previous studies, most of the template switching events were observed between an L1 RNA or a *trans*-mobilized cellular mRNA on one end and U6 or U6atac snRNA on the other end (Buzdin et al. 2003; Garcia-Perez et al. 2007) (table 1). Remarkably, in our study, two-thirds of the snRNA sequences associated with repeats are U6 or U6atac (21 copies; table 1). Out of them, 19 copies present TSD at their extremities and the insertion site contains the L1 consensus cleavage site (table 1 and supplementary table S1, Supplementary Material online).

We observed seven U5 snRNA copies associated with retrotransposed sequences (L1, SVA, and a sequence of unknown origin; table 1 and supplementary table S1, Supplementary Material online). From these, three seem to represent events of template switching (sequences # 5, 8, and 9 in fig. 1*B*). For sequences # 5 and 8, the reverse transcription started from an L1 RNA and then switched to U5 snRNA. For sequence # 5, a subsequent *Alu* insertion at the 3′-end of the L1 induced a short genomic deletion at the integration locus including the 3′-TSD. However, we were able to fully characterize the original insertion site by comparing with the orthologous site in the *Macaca mulatta* genome (for which the *Alu* insertion is not present, data not shown). For sequence # 9, the 3′-flanking sequence between the TSD (15 bp) and the U5 sequence is of unknown origin. For the three U5 chimeras described above, it is worth noticing that the U5 segments are 3′-truncated (positions 83, 78, and 98, respectively, over a 116 nt sequence), as it was previously observed for sequence #8 and for U3 snoRNA chimeras (Buzdin et al. 2003). Thus, we suggest that the template switching from L1 RNA to U5 snRNA occurs mainly, if not only, internally. Such internal initiation during template switching has never been observed for the formation of U6/L1 chimeras.

The remaining four U5 copies of this group are associated with L1 or SVA in the opposite transcriptional orientation, in between the TSD, suggesting for each a single mobilization event (sequences # 3, 4, 6, and 7 in fig. 1*B*); U5 sequences are also 3′-truncated (positions 36, 48, 43, and 46, respectively). Interestingly, when looking at these insertions using the U5 transcriptional orientation, we noticed that the cleavage sites differ from the L1 EN consensus (fig. 1*B*, Cleavage). However, when considered from the L1 transcriptional orientation, each cleavage site corresponds to the L1 EN cleavage consensus (fig. 1*B*, Remarks (*)). This suggests that U5 snRNA can be recruited at the top strand cleavage of the insertion site, a mechanism previously described for L1 and named twin priming (Ostertag and Kazazian 2001). Again, it also indicates that the reverse transcription can be initiated internally within the sequence of U5 snRNA (fig. 1*A*[*c*′] and [d′]). In support of this model, we also observed sequence complementarities between the U5 segment and the insertion site (fig. 1*B*, Junction). Complementarity may have facilitated the internal initiation of reverse transcription on the top strand, as is thought to be the case for twin priming (Ostertag and Kazazian 2001; Gilbert et al. 2005). Noticeably, if we look at the predicted secondary structure of the U5 snRNA, all four truncations are located on the same single-stranded region, a loop domain implicated in the interaction with upstream and downstream exons during the splicing process (see review [Patel and Steitz 2003]).

The U1/L1, U2/L1, and one U6/L1 chimeras observed in this study present the same characteristics as described above for the last four U5 sequences (table 1 and fig. 1*B*, sequences # 1, 2, and 10). They are 3′-truncated and inserted in inverse transcriptional orientation of L1. Thus, the mechanism of inverted U/L1 chimera formation seems to differ from the standard U6/L1 template-switching model, resembling more closely the twin priming model (fig. 1*A*).

Finally, only two copies of U6atac were found to be associated with *Alu* sequences. Previously, examples of U6/Alu chimeras have been reported (Buzdin et al. 2003; Garcia-Perez et al. 2007). However, their formation was not experimentally reproduced, suggesting that the frequency of such an event is very low (Garcia-Perez et al. 2007). Here, between the two copies of U6atac/*Alu*, only one potentially represents a true chimera formed by template switching, as it is flanked by a TSD of 14 bases. For the other example, no TSD was found and an extra adenine is present at the junction between U6atac and *Alu*. This suggests that the resultant structure could have been generated after two independent retrotransposition events. Such rare occurrence of snRNA pseudogenes associated with *Alu* sequences may be due to specificities of *Alu* insertion mechanism. Indeed, we can speculate on the role of ORF1p in template switching as it has been shown that *Alu* can retrotranspose without the presence of ORF1p (Dewannieux et al. 2003; Garcia-Perez et al. 2007).

Overall, among the 30 snRNA copies associated with retrotransposons, 23 represent sequences formed by template switching, and the 7 other sequences could have been formed by twin priming. Interestingly, these results confirm that template switching might be restricted to U6 and U6atac snRNAs (Garcia-Perez et al. 2007) as they represent most of the validated template switching insertions. Moreover, it has been recently shown that only U6 snRNA was enriched in L1 RNP immunoprecipitation pullouts (Taylor et al. 2013), indicating a peculiar relationship with L1 retrotransposition machinery. At this stage of the analysis, we can list multiple features of U6 and U6atac to explain why they may be favored in chimera formation by template switching. First, they have a different transcription mode compared with other snRNAs. Indeed, U6 and U6atac are transcribed by the RNA Polymerase III whereas the others involve the RNA Polymerase II (Hernandez 2001). In consequence, U6 and U6atac snRNAs are the only two ending with a stretch of uraciles, due to the presence of an RNA pol III terminator sequence (i.e., a stretch of 4–5 thymines). Second, U6 and U6atac have peculiar subcellular localization. They are located in the nucleus, whereas the other snRNAs shuttle to the cytoplasm for maturation before returning to the nucleus where splicing occurs. Moreover, U6 and U6atac transcripts undergo maturation in specific nuclear compartments, such as the nucleolus (see review [Kiss 2004]), where L1 proteins may be transiently located (Goodier et al. 2004). Third, U6 and U6atac also share specific protein partners, such as Lsm proteins (Like-Sm proteins) that bind the uracil end of both transcript (Matera et al. 2007), that could help interaction with the L1 retrotransposition complex. Finally, U6 and U6atac share a common role in the splicing reaction as they take part in equivalent snRNPs in the major and minor spliceosomal complexes, respectively (Patel and Steitz 2003). Each of these specificities, separately or together, may be involved in favoring U6 and U6atac mobilization by template switching resulting in chimera formation.

In a previous publication, a small number of snRNA copies were found associated with LTR retrotransposons (Giles et al. 2004). In some cases, the copies were amplified in independent events from the retroviral insertions. However, in other cases, the snRNA pseudogene formation may have occurred concomitantly with the LTR retrotransposon insertions. Nevertheless, these insertions are ancient, with a sequence divergence higher than the parameters established in this study. Thus, snRNA mobilization by LTR retrotransposons does not appear to have occurred in a more recent time. It also correlates with the fact that LTR retrotransposons seem to be no longer active in the human genome.

### Polyadenylated snRNA

We retrieved many snRNA sequences followed by a poly(A) or an A-rich tract (15% of the total). They are highly represented for U1 (54% of all U1 analyzed sequences) but very rare for U2 and U5 (0 and 1.5%, respectively). The majority of these poly(A)-extended pseudogenes is flanked by variable size TSD (70% of the overall copies) and the consensus cleavage site resembles that of L1 EN (not shown). We propose two models to explain the formation of such processed pseudogenes in the genome. First, they could be formed by early template switching during reverse transcription from the poly(A) tail of an L1 RNA to the snRNA. This scenario seems possible for U6/poly(A) and U6atac/poly(A) structures as we have observed chimeras between L1 and the two snRNAs (table 1). However, as other snRNA (particularly U1 and U4) form chimeras with L1 extremely rarely, template switching may not be the mechanism involved in the formation of U/poly(A)-processed pseudogenes. Indeed, out of 33 U1 sequences, 18 present a 3′ poly(A) extremity (of which 13 have TSD), and no U1/L1 chimeras generated by template switching were observed.

Thus, for the second model, poly(A)-extended pseudogene formation could occur by the direct recruitment of already polyadenylated snRNAs by the L1 retrotransposition machinery. Early work on snRNA pseudogenes already identified sequences followed by A-rich tracts flanked by TSD, and an atypical polyadenylation mechanism prior integration through retrotransposition was proposed (Van Arsdell et al. 1981; Denison and Weiner 1982). More recently, such types of snRNA structures have been identified in cells. They originate from the early step of the nuclear small RNA surveillance/turnover mechanism, which consists of poly(A) extension at the 3′-end of the snRNA. This labeling directs them to the nuclear exosome for degradation (see review [Houseley et al. 2006]). Thus, we can suggest that the L1 retrotransposition complex, or at least ORF2p, can be associated with such polyadenylated RNAs and initiate retrotransposition from these templates. Moreover, aberrant snRNA transcripts, including prematurely terminated snRNAs, are also poly(A) extended by the RNA surveillance machinery. In agreement with this, we identified 3′-truncated snRNA pseudogenes that are followed by a poly(A) tract and flanked by TSD (2 cases for U1, 1 for U5, and 2 for U6; all included in table 1, Poly(A)).

**Fig. 2.**
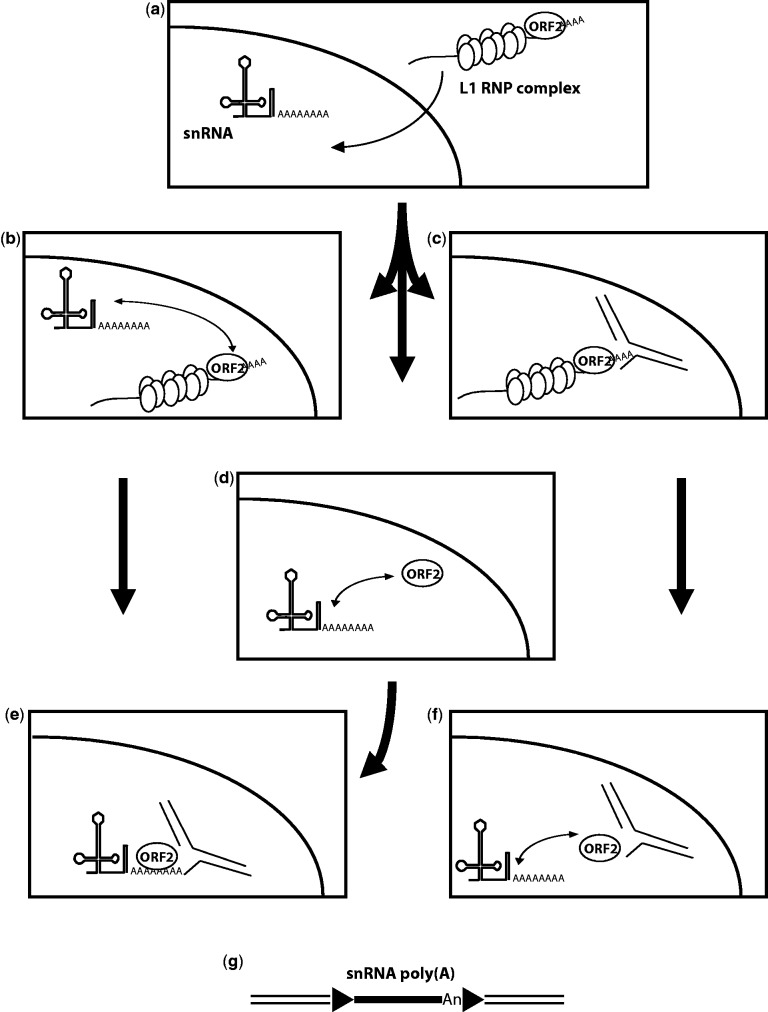
Formation of polyadenylated snRNA pseudogenes. The L1 RNP complex is constituted by ORF1p homotrimers (vertical ovals), ORF2p (horizontal oval), and L1 RNA (wavy line with poly(A) tail). Cleaved genomic DNA target is represented by interrupted black lines. (*a*) The polyadenylated snRNA is present in the nucleus and the L1 RNP complex formed in the cytoplasm enters the nucleus. (*b*) ORF2p dissociates from the L1 RNP complex and is then associated with the polyadenylated snRNA. (*c*) L1 RNP complex cleaves the target site (first step of TPRT). (*d*) Free nuclear ORF2p binds to polyadenylated snRNA. (*e*) The RNP formed by ORF2p and the polyadenylated snRNA from panel (*b*) or (*d*) initiates TPRT. (*f*) L1 RNA dissociates from the L1 RNP depicted in panel (*c*) at the target site, and polyadenylated snRNA is associated with the free ORF2p still present at the target site. (*g*) Resolution of the initiated TPRT from panel (*e*) or (*f*). In panel (*g*), arrowheads represent TSD.

This second model of U/poly(A) chimera formation would suggest that snRNAs could be recruited in the nucleus and multiple putative scenarios are possible (fig. 2). In the first scenario, the L1 RNP complex (containing at least both L1 proteins and L1 RNA) enters the nucleus and the L1 RNA is replaced by a polyadenylated snRNA to form the new RNP complex that will undergo insertion by TPRT (fig. 2*a*, *b*, *e*, and *g*). In the second scenario, the L1 RNP complex initiates the first step of TPRT (i.e., first strand cleavage by the L1 EN), and then looses its L1 RNA template before initiating reverse transcription. It can then recruit a polyadenylated snRNA present in the nucleus to initiate reverse transcription and finalize a retrotransposition event (fig. 2*a*, *c*, *f*, and *g*). Finally, the third possibility is that free ORF2p could exist in the nucleus, either because it escaped the RNP formation and is addressed to the nucleus, or upon dissociation of L1 RNP complex, after finishing a first insertion. The free ORF2p can recruit polyadenylated snRNA present in the nucleus to form an RNP complex and initiate a new retrotransposition event (fig. 2*a*, *d*, *e*, and *g*).

Interestingly in the human genome, a previous report highlighted the presence of pre-tRNA retropseudogenes mediated by the L1 machinery (Schmitz et al. 2004). No pre-tRNA has ever been found in the cytoplasm of vertebrates and the three enzymes (EN, ligase and 2′-phosphotransferase) implicated in tRNA splicing seem to act in the nucleus (see review [Hopper and Shaheen 2008]). Thus, these observations further support the possible nuclear recruitment of cellular RNA.

### 3′-Truncated snRNA Pseudogenes Are Mobilized by L1

We finally observed that the majority of the characterized genomic snRNA copies was not associated with any retrotransposon sequence and was 3′-truncated (65% of the total). Indeed, they can represent between 30% and 98% of all sequences retrieved depending on the snRNA.

**Fig. 3.**
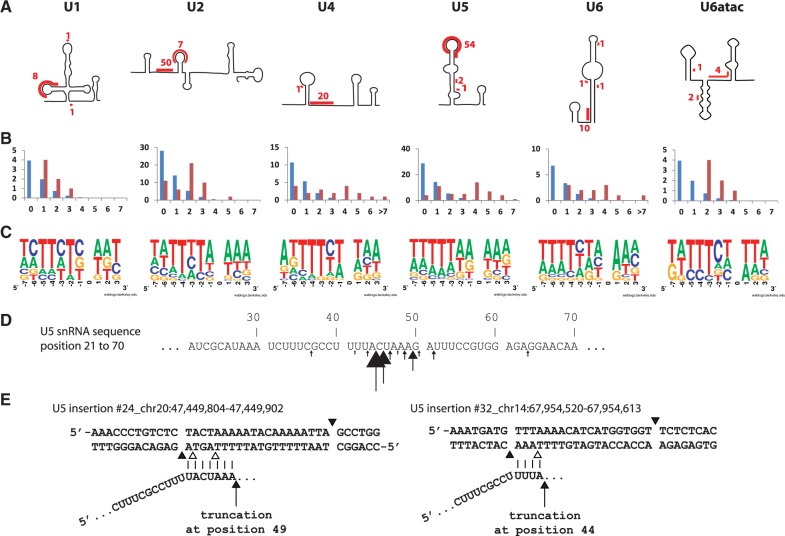
3′-truncated snRNA pseudogenes. (*A*) Schematic representation of snRNA secondary structures (based on Rinke et al. 1985 and Patel and Steitz 2003). Segment of sequences where truncation occurred and the number of occurrences are highlighted in red. (*B*) Distribution of the number of nucleotide homologies at the 3′-junction (*Y*-axis, number of occurrences; *X*-axis, number of nucleotides). The blue bars represent the expected random distribution, and red bars, the observed distribution. (*C*) Representation of the consensus cleavage site using WebLogo (Crooks et al. 2004). (*D*) Segment of the U5 snRNA where the majority of truncations occurred. Arrows are pointing truncation sites with size proportional to the occurrence of truncation events. (*E*) Two examples of U5 snRNA 3′-truncation. Vertical bars represent sequence homology between genomic DNA and U5. Black arrows indicate putative truncation points on the snRNA sequence. Black arrowheads delimitate the considered TSD. Empty arrowheads correspond to the L1 EN consensus cleavage sites potentially used by ORF2p to generate the first genomic DNA nick.

For each of the snRNA analyzed, between 70% and 100% of the truncated sequences are flanked by TSD, suggesting that they are amplified by a retrotransposition-mediated mechanism. To confirm this hypothesis, we further analyzed all truncated copies of each snRNA. Here, we first observed that truncations were not randomly distributed throughout the snRNA sequence but were grouped in specific short segments. Based on snRNA-predicted secondary structures (Rinke et al. 1985; Patel and Steitz 2003), truncations seem to occur almost always at a single-stranded RNA segment (fig. 3*A*). Then, we observed sequence complementarities at the 3′-junction between the truncated sequences and the insertion site (fig. 3*B*). Finally, we were able to build a consensus cleavage site for each snRNA group, using all insertion sites with identified TSD. They all resemble the L1 EN preferential cleavage site 5′-TTTT/A (fig. 3*C*). For U2, U5, and U6, we observed a shift of one or two adenosines to the 5′-segment of the cleavage site compared with the L1 consensus (fig. 3*C*). This could be explained by truncation occurring in a particular short segment of the U sequences (example for U5; fig. 3*D*). All these segments are purine rich, which allows a short pairing with the pyrimidine rich single-stranded genomic DNA at insertion site generated by the L1 EN cleavage. This pairing may help initiation of reverse transcription by L1 ORF2p (Monot et al. 2013; Viollet et al. 2014). A closer look at each segment implicated in the pairing showed that purine stretches on the snRNAs are preceded by a thymine (except for U1 where the purine-rich segment is interrupted by two cytosines). As we always consider the longest identical sequence present on each side of the insertion to define TSD, the thymine is included in the TSD and thus included in the 5′-segment of the cleavage site (two examples are shown in fig. 3*E*).

All the observations above lead us to propose that the L1 retrotransposition machinery mediates the formation of 3′-truncated snRNA copies by template choice, that is initiating reverse transcription on the complemented RNA directly. Single-stranded RNA segments leave the opportunity for the snRNA to pair with the single-stranded DNA generated by the L1 EN cleavage at the insertion site, and facilitate the initiation of reverse transcription by L1 ORF2p. A similar model has been suggested for the formation of 3′-truncated snRNA (Denison and Weiner 1982) and tRNA retrospeudogenes, named “tailless retropseudogenes” (Schmitz et al. 2004).

**Fig. 4.**
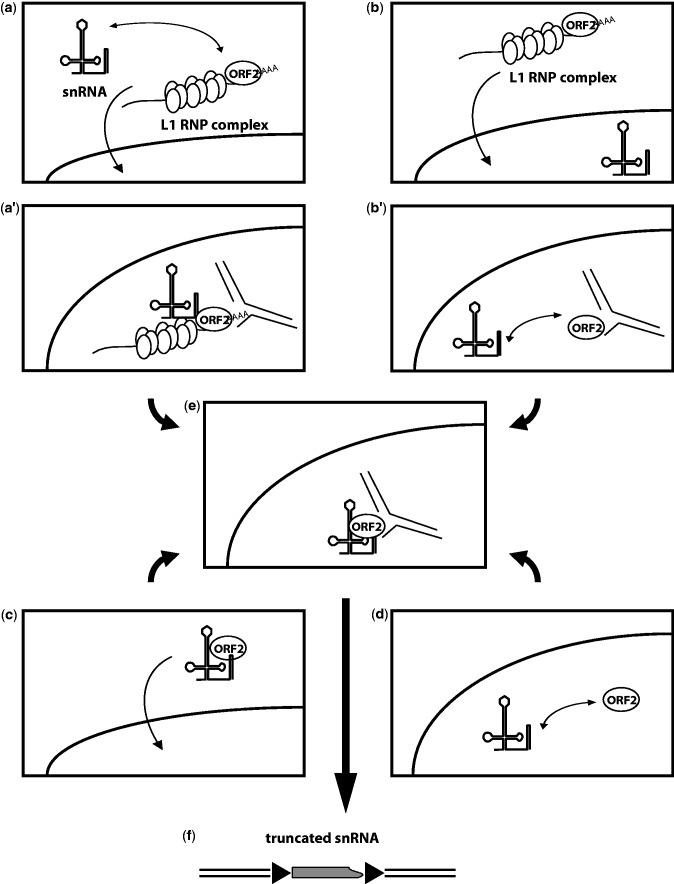
Formation of 3′-truncated processed snRNA pseudogenes. The L1 RNP complex is constituted by ORF1p homotrimers (vertical ovals), ORF2p (horizontal oval), and L1 RNA (wavy line with poly(A) tail). Cleaved genomic DNA targets are represented by interrupted black lines. (*a*) snRNA present in the cytoplasm can be associated with the cytoplasmic L1 RNP complex and, together, enters the nucleus. (*a*′) The snRNA L1 RNP complex initiates TPRT, and can lose the L1 RNA (see panel *e*). (*b*) L1 RNP complex formed in the cytoplasm enters the nucleus. (*b*′) The L1 RNP complex initiates TPRT, and then loses its L1 RNA which is “replaced” by a nuclear snRNA. (*c*) A cytoplasmic RNP complex is formed by the association of free ORF2p and an snRNA, and then enters the nucleus. (*d*) In the nucleus, free ORF2p associates with a nuclear snRNA to form an RNP complex. (*e*) RNP complex from either panel (*a*′), (*b*′), (*c*), or (*d*) initiates (for *c* and *d* only) and process TPRT. (*f*) Resolution of the TPRT from panel (*e*). In panel (*f*), arrowheads represent TSD.

Similar to the mechanism proposed for the formation of U/poly(A) chimeras (fig. 2), several models could explain the recruitment of snRNAs to form 3′-truncated processed pseudogenes (fig. 4). In the first model, snRNAs could be associated with the retrotransposition complex in the cytoplasm and be transported to the nucleus where insertion occurs. The complex initiates reverse transcription internally on the snRNA (fig. 4*a*, *a*′, *e*, and *f*). In a second model, snRNA and the retrotransposition complexes could enter the nucleus independently. The L1 RNP complex subsequently initiates the first step of TPRT (i.e., target cleavage), and then loses its original RNA template before the initiation of reverse transcription. ORF2p would then recruit an snRNA present in the nucleus to finalize the retrotransposition event (fig. 4*b*, *b*′, *e*, and *f*). In a third alternative model, ORF2p would form an RNP complex with an snRNA in the cytoplasm, similar to the model proposed for *Alu* retrotransposition complex formation (Boeke 1997; Dewannieux et al. 2003). This complex enters the nucleus and generates a retrotransposition event (fig. 4*c*, *e*, and *f*). Finally, as proposed earlier, free ORF2p could be found in the nucleus. This protein could form a nuclear RNP complex with an snRNA and then undergo retrotransposition (fig. 4*d*–*f*).

### snRNA-Processed Pseudogenes Are Common to All Mammalian Genomes

We further aimed to analyze the U6 snRNA pseudogene distribution in all placental mammals with available assembled genomes (39 species; table 2). For this purpose, we developed a bioinformatics pipeline named ProRNAScan to analyze small RNA pseudogenes. This program arranges highly similar nucleotide sequences (identified by BLAST) in groups based on their structure and flanking sequences (the same four groups described in table 1). The pipeline is set by default with the selective parameters established for the human U6 snRNA analysis (i.e.*,* 97.5% identity to the referring sequence and at least 26 nucleotides in length). We have combined the results for each genome analyzed in table 2.

**Table 2.** Distribution of Processed U6 snRNA Sequences in Vertebrate Genomes.

Common Name	Species Name	Total Hits^a^	Hits Selected^b^	Hits Analyzed (TSD)^c^	Alone	Repeat	Poly(A)	3′-trunc
Xenopus	*Xenopus tropicalis* (75)	48	18	5 (0)	5	0	0	0
Chicken	*Gallus gallus* (74)	41	4	4 (0)	4	0	0	0
Turkey	*Meleagris gallopavo* (74)	32	4	1 (0)	1	0	0	0
Zebra finch	*Taeniopygia guttata* (74)	10	3	2 (0)	2	0	0	0
Lizard	*Anolis carolinensis* (74)	192	52	41 (20*)	0	16	0	25
Platypus	*Ornithorhynchus anatinus* (74)	412	70	47 (*)	7	40	0	0
Tasmanian devil	*Sarcophilus harrisii* (74)	297	16	5 (1)	4	0	0	1
Wallabi	*Macropus eugenii* (74)	252	7	4 (2)	2	2	0	0
Opossum	*Monodelphis domestica* (76)	721	16	9 (*)	3	5	0	1
Sloth	*Choloepus hoffmanni* (74)	253	15	8 (5)	2	4	2	0
Armadillo	*Dasypus novemcinctus* (74)	604	218	174 (166)	6	136	30	2
Tenrec	*Echinops telfairi* (74)	263	72	65 (60)	7	9	47	2
Elephant	*Loxodonta africana* (74)	779	20	15 (11)	4	10	1	0
Hyrax	*Procavia capensis* (74)	257	9	7 (2)	2	1	3	1
Hedgehog	*Erinaceus europaeus* (74)	258	93	72 (56)	9	11	49	3
Shrew	*Sorex araneus* (74)	274	111	79 (62)	6	49	21	3
Microbat	*Myotis lucifugus* (74)	918	126	111 (101)	3	10	77	21
Megabat	*Pteropus vampyrus* (74)	296	9	4 (3)	1	0	0	3
Horse	*Equus caballus* (74)	416	27	21 (14)	5	11	2	3
Cat	*Felis catus* (74)	1,918	322	279 (262)	5	17	21	236
Dog	*Canis familiaris* (67)	2,849	454	415 (392)	5	26	68	316
Panda	*Ailuropoda melanoleuca* (74)	469	32	24 (20)	5	5	14	1
Ferret	*Mustela putorius furo* (78)	2,519	587	504 (500)	5	11	48	440
Dolphin	*Tursiops truncatus* (74)	294	43	31 (20)	7	10	14	0
Pig	*Sus scrofa* (74)	905	54	39 (34)	5	20	7	7
Cow	*Bos taurus* (74)	1,099	148	122 (103)	15	54	34	19
Sheep	*Ovis aries* (78)	1,085	171	149 (125)	8	100	24	17
Alpaca	*Vicugna pacos* (74)	310	48	36 (30)	2	6	27	1
Mouse lemur	*Microcebus murinus* (73)	287	68	49 (44)	2	8	38	1
Bushbaby	*Otolemur garnettii* (75)	2,457	162	120 (109)	7	25	71	17
Tarsier	*Tarsius syrichta* (73)	256	21	12 (8)	1	6	4	1
Marmoset	*Callithrix jacchus* (73)	1,963	179	128 (117)	12	27	60	29
Macaque	*Macaca mulatta* (73)	1,344	73	48 (45)	2	13	16	17
Olive baboon	*Papio anubis* (78)	1,370	65	52 (45)	4	9	17	22
Vervet-AGM	*Chlorocebus sabaeus* (78)	1,385	45	30 (24)	4	9	5	12
Orangutan	*Pongo abelii* (73)	1,422	39	20 (15)	4	7	2	7
Gorilla	*Gorilla gorilla* (73)	1,401	47	19 (13)	4	8	4	3
Chimpanzee	*Pan troglodytes* (73)	1,466	50	24 (20)	5	9	2	8
Human	*Homo sapiens* (73)	1,515	55	37 (29)	5	13	6	12
Gibbon	*Nomascus leucogenys* (73)	1,455	71	46 (40)	3	9	8	26
Tree shrew	*Tupaia belangeri* (74)	279	80	66 (50)	14	21	24	7
Pika	*Ochotona princeps* (74)	306	136	78 (63)	5	41	24	3
Rabbit	*Oryctolagus cuniculus* (74)	1,140	181	133 (118)	11	30	33	59
Guinea Pig	*Cavia porcellus* (74)	1,416	73	54 (47)	7	29	9	9
Kangaroo rat	*Dipodomys ordii* (74)	276	47	18 (14)	3	12	3	0
Mouse	*Mus musculus* (74)	904	159	147 (128)	7	109	15	16
Rat	*Rattus norvegicus* (74)	1,010	295	241 (217)	7	50	33	151
Squirrel	*Ictidomys tridecemlineatus* (76)	829	44	29 (24)	3	6	10	10

Note.—The first and second columns indicate the common and scientific names of the analyzed genomes, respectively. The release number of the sequenced genome used in this study is indicated in parenthesis after the scientific name.

^a^Number of sequences found by BLAST in the corresponding genome.

^b^Number of analyzed sequences after applying selective parameters (see Materials and Methods section).

^c^Effective number of unique sequences characterized (see Materials and Methods section). Numbers in parenthesis (TSD) gives the number of sequences with identified TSD. In three genomes, TSD of less than nine nucleotides was found for sequences associated with repeats but was not included (noted by an asterisk). The next columns give the distribution of copies depending on the identified associated sequences (Alone, sequences not associated with repeats; Repeat, sequences associated with retrotransposon; Poly(A), sequences with an A-rich 3′-extremity; 3′-trunc, 3′-truncated copies).

**Fig. 5.**
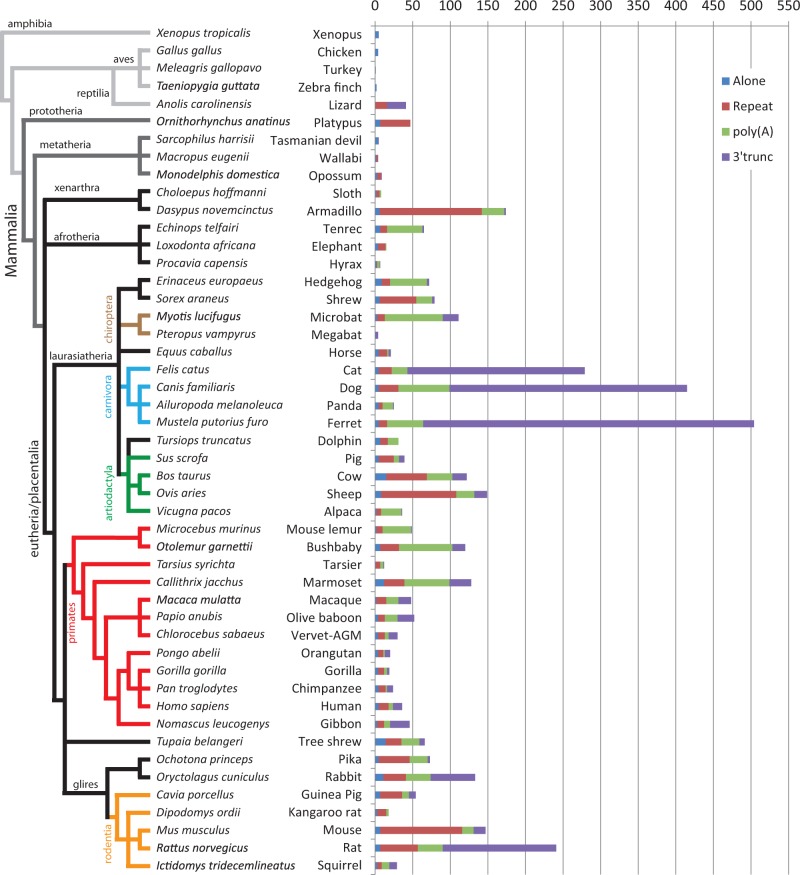
Number and distribution of U6 snRNA sequences in vertebrate genomes. The left side of the figure is a schematic representation of the phylogenetic tree of the species analyzed in this study obtained from the Ensembl project website (http://www.ensembl.org/info/about/speciestree.html, last accessed March 20, 2015). Black branches regroup the eutheria clade and some of the orders are highlighted by specific colored branches: Chiroptera in brown, carnivora in blue, artiodactyla in green, primates in red, rodentia in orange. The right side of the figure presents the accumulated numbers of each group of analyzed U6 snRNA sequences in 48 vertebrate genomes, reflecting the “quantitative” and “qualitative” variability of recruitment through retrotransposition.

We first observed a wide variability in terms of copy number depending on the genome analyzed (table 2). Using BLAST default parameters in ProRNAScan, the number of U6-derived sequences ranged from 253 for *Choloepus hoffmanni* (sloth) to 2,849 for *Canis familiaris* (dog). This variation in U6 pseudogene occurrences does not always correlate with the phylogenetic relationship existing among species (fig. 5). For example, we observed a large variation of U6 occurrences among rodent genomes (table 2, and orange branches in fig. 5). However, in primates, and particularly in apes, U6 occurrences vary less (table 2, and red branches in fig. 5). Using our pipeline, we next classified the most conserved sequences in the four predefined groups. The results demonstrated that L1 is capable of mobilizing U6 snRNA in all mammalian genomes analyzed (table 2 and fig. 5). We also observed that the proportion of each group of U6 snRNA pseudogenes varies widely between genomes (fig. 5). In order to validate the differential distribution of the groups among genomes, we performed a Fisher’s exact test comparing each of the 48 genomes with each other (including noneutherian species; supplementary table S2, Supplementary Material online). We divided this analysis by comparing genomes within the same phylogenetic group. If we consider primates, for which there is the largest number of genomes available, and particularly apes, we observed that most of *P* values are greater than 0.01. This indicates that the compared data sets are not statistically different (fig. 5 and supplementary table S2, Supplementary Material online). Therefore, we can conclude that L1 retrotransposition dynamics is similar among apes (reflected by the distribution of U6 pseudogene structures). However, L1 dynamics clearly changes starting from marmoset, a New World monkey, to mouse lemur (red table in supplementary table S2, Supplementary Material online). We next considered other phylogenetic groups, such as rodentia, cetartiodactyla, carnivora, and chiroptera orders (in orange, green, blue, and brown, respectively, inside of supplementary table S2, Supplementary Material online). The results of the Fisher’s exact test suggest that L1 retrotransposition dynamic varies inside each phylogenetic group (fig. 5 and supplementary table S2, Supplementary Material online). However, the number of available genomes and the representation of each phylogenetic order are currently not sufficient to make definitive conclusions about the relationship between phylogeny and retrotransposition dynamics. Nevertheless, if we compare all placental mammalian genomes, the variability of U6 pseudogene formation is not only quantitative but also qualitative, as different mobilization pathways can be used more or less frequently in a given genome (fig. 5). Based on this global observation, we suggest that the L1-mediated genomic amplification of the U6 snRNA can serve as an indirect read-out for L1 dynamics among mammalian genomes. Two major hypotheses can be proposed to explain the observed variability. First, as L1s evolved independently in each genome after the divergence of mammals, it is possible that their ability to recruit cellular RNAs has also evolved and now use or favors different pathways. Alternatively, evolution of cellular factors interacting with L1s may impact retrotransposition leading to variable mobility dynamics in each genome. A global view would suggest that both hypotheses are valid and the combination of the two contributed to the observed copy number variability of U6 snRNA in genomic DNA.

### snRNA-Processed Pseudogene Formation Is Not Specific to L1 Activity

Finally, using our bioinformatics pipeline, we have expanded our analysis to other vertebrate genomes, from marsupial to amphibian. We first looked in *Metatheria*, the other clade of the *Theria* subclass for which three genomes are available (*Monodelphis domestica*, *Macropus eugenii,* and *Sarcophilus harrisii*). We detected only a small number of U6 snRNA copies per genome that fulfilled the selective parameters (less than 10 per genome; table 2). These low numbers would suggest a loss of L1 activity in marsupials as it seems to be the case for the Tasmanian devil (Gallus et al. 2015). However, L1 seems to be active in some *Metatheria* as it has been able to efficiently amplify in *trans* the evolutionary recent nonautonomous SINE-1 element (in *M. domestica*; Gentles et al. 2007). Nevertheless, we were able to find sequences for each group defined above, U6-L1 chimeras, U6-polyA extended sequences, and 3′-truncated elements. Interestingly, in opossum only, we found one 5′-truncated U6 sequence associated with an RTE-like element (RTE_Mdo) and another, also 5′-truncated, associated with an RTESINE1 element. The latter, RTESINE1 is a recent SINE element believed to be mobilized by RTE_Mdo (Nilsson et al. 2010). However, no obvious TSD was found for either chimeras, and thus we cannot definitively conclude on the formation of chimeras involving a non-L1 clade element in the opossum genome.

In Platypus, a *Prototheria*, we detected a higher number of conserved U6 snRNA sequences (table 2). The U6 copies are either alone, most likely corresponding to active genes, or associated with repeats flanked by TSD. Notably, unlike placental mammals, the repeats are LINE-2 (L2) or Mon-1 (a SINE element believed to be mobilized by L2; Gilbert and Labuda 1999). Thus, U6 chimeras generated by retrotransposition using template switching mechanism are not a specificity of LINEs from the L1 clade. Interestingly, in contrast to L1 proteins which follow a relaxed model as they are thought to nonspecifically mobilize RNAs through their poly(A) tract (Esnault et al. 2000; Wei et al. 2001; Roy-Engel et al. 2002; Dewannieux et al. 2003; Dewannieux and Heidmann 2005), L2 proteins follow a stringent model by binding specifically to an RNA structure present in the 3′-UTR of L2 (Kajikawa and Okada 2002; Hayashi et al. 2014). This implies that cellular RNAs have to acquire the specific RNA segment to allow their mobilization by the L2 machinery. Indeed, a proposed mechanism for the acquisition of the 3′-segment of an LINE by a SINE is template switching (Gilbert and Labuda 1999). Therefore, in genomes with active stringent LINEs (e.g., in platypus), it is rare to detect processed pseudogenes. Similarly, the requirement of an L2-specific sequence for mobilization can explain why we do not detect U6 associated with poly(A) or 3′-truncated copies, as observed when a “relaxed” LINE (i.e.*,* L1) is involved. Other notable differences have been found. First, almost all of the U6-L2 chimeras in the platypus genome have a 5′-truncated U6 segment upstream of a short L2 sequence (only one U6 sequence is full length). Second, and consistent with the insertion mechanism of L2 which is initiated from a simple repeat at the 3′-end of the sequence (here TGAA) (Ichiyanagi and Okada 2008), only small TSDs are found (from 1 to 9 bp) for 72.5% of the 40 chimeras.

We next expanded our analysis to reptiles by screening the lizard genome (*Anolis carolinensis*). We found 43 U6 copies that reached our selective criteria and were able to classify all of them into two of the four predefined groups:1) Associated with repeats and 2) 3′-truncated (table 2). No original U6 gene was found (table 2, Alone), most likely because the genome assembly is still incomplete. The most striking result from the *A. carolinensis* genome is the finding of U6 chimeras with three different LINE families, each belonging to a different clade. Indeed, we found 3 U6 copies with LINE-1-like (L1 Acar), 3 with LINE-2-like (L2 Acar), and 3 more with RTE-like (RTEX-2 Acar) LINE elements. This observation demonstrates that template switching from LINE RNA to U6 snRNA during the process of retrotransposition is not specific to LINE-1 clade elements but is a more general property of autonomous non-LTR retrotransposons. Moreover, we also found six U6 chimeras with AnolisSINE2 sequences, which is believed to be mobilized by the L2 Acar of the lizard genome (Piskurek et al. 2009). Thus, as in platypus genome with Mon-1, AnolisSINE2 can form chimeras with U6 by a template switching mechanism.

In birds, for which three genomes are available (*Gallus gallus*, *Meleagris gallopavo,* and *Taeniopygia guttata*), only few copies of U6 reached the selective criteria (three or four sequences per genome). Nevertheless, in turkey (*Me. gallopavo*), we identified one 5′-truncated U6 copy associated with a CR1-like element. The full characterization of this copy was not possible due to the presence of an unsequenced gap (succession of Ns) within the CR1 sequence, and thus we could not definitively conclude on the structure of the chimera.

Finally, in *Xenopus tropicalis*, only 5 of 32 sequences had more than 97.5% identity to the reference U6 gene. These five copies are full length and most likely represent active copies. Thus, in this particular genome, we have not found evidence of U6 snRNA-processed pseudogenes.

## Conclusion

**Fig. 6.**
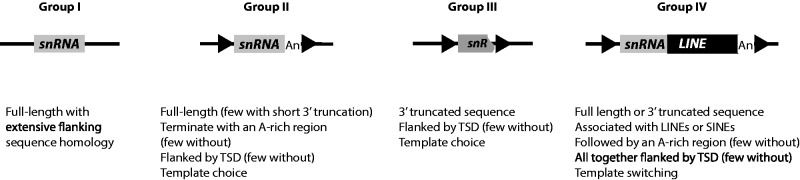
Proposed classification for genomic copies of snRNAs. Dispersed copies are classified into four groups. Gray rectangles represent snRNA gene or pseudogene, gray broken rectangle represents 3′-truncated snRNA pseudogene, and black rectangle represents non-LTR retrotransposons (SINE or LINE). Black arrowheads represent TSD. In Group IV, LINEs are usually 5′-truncated. Few cases of twin priming between an snRNA and an LINE RNA have generated pseudogene chimeras and are included in Group IV. In this figure, and compared with figure 1 in Denison and Weiner (1982), Group I corresponds to Class I, Group II associates Class II and III, Group III is Class IV. Group IV was not described originally.

In the early 1980s, when sequencing was at its beginning, four classes of snRNA pseudogenes were identified. Evidence of the use of an RNA intermediate for pseudogene formation was already proposed for three of these classes, even though little was known about retrotransposition mechanisms (Van Arsdell et al. 1981; Denison and Weiner 1982; Van Arsdell and Weiner 1984). Today, we can amend this classification. We propose to divide snRNA genomic copies into four groups (fig. 6). Group I corresponds to duplications of snRNA genes and their flanking sequence, and was previously defined as Class I (Denison and Weiner 1982). Group II includes previous Class II and III pseudogenes and corresponds to processed snRNA pseudogenes that generally end with an A-rich tail and are flanked by TSD. Group III corresponds to Class IV in the previous classification, and represents processed pseudogenes that are heavily 3′-truncated and flanked by TSD. To generate pseudogenes from Groups II and III, the proposed mechanism of amplification is that reverse transcription initiates directly on the snRNA by a template choice. Finally, Group IV corresponds to snRNA pseudogenes that form chimeras with a non-LTR retrotransposon. Their mechanism of amplification has been described previously and is known as template switching (Buzdin et al. 2002; Garcia-Perez et al. 2007). In a few cases, we have observed a new form of chimera for which the snRNA sequence is in the opposite transcriptional orientation relative to the retrotransposon sequence. Here, we suggest a model to describe the steps leading to such chimeras (fig. 1). Our model involves twin priming at the insertion site, a mechanism originally suggested to explain the inverted/deleted forms of L1 copies found in mammalian genomes (Ostertag and Kazazian 2001). Because such type of chimera is mainly observed with U5 snRNA, we can speculate that the sequence, structure, or function of U5 may be responsible for its mobilization by twin priming.

Our study reinforces the notion of the existence of two major insertion pathways for pseudogene formation through retrotransposition: By template choice or by template switching. Interestingly, in either case, our data suggest that reverse transcriptional initiation can occur either at the 3′-extremity or internally on the cellular RNA molecule. Here, we propose that at least two pathways may exist for the retrotransposition complex to recruit snRNAs, one in the nucleus and the other in the cytoplasm (figs. 2 and 4). However, further experimental investigations are required to answer the remaining questions, such as where and how cellular RNAs are recruited by the LINE retrotransposition complex.

The study shows that, in the human genome, snRNA copies are not equally distributed in the groups defined above. Indeed, most of the U2 copies are in Group III whereas most of the U5 copies are in Group II (table 1, fig. 6). This suggests the existence of variable affinities with the retrotransposition machinery and potentially variable recruiting steps for each snRNA. Moreover, the mobilization dynamics of a particular snRNA (U6 snRNA in this study) is highly variable when several mammalian genomes are compared. Once again, this suggests that retrotransposition pathways may be multiple and specific to each genomic environment. This can include different interacting cell factors and different active LINEs. These discrepancies among mammalian genomes result in variable processed pseudogene mobilization efficiencies and structures. Furthermore, these differences could serve as an indirect read-out of L1 activity among mammalian genomes. In agreement to this, Cantrell et al. (2008) have shown that L1 activity was lost in megabats. Indeed, we observed a drop of the U6 copy number in the megabat genome (table 2, Total Hits) as well as a drastic decrease of younger sequences compared with other mammals (table 2, Hits Selected, and fig. 5). Thus, a simple screen of U6 genomic copies on a newly sequenced mammalian genome should indicate the status of L1 retrotransposition activity. Accordingly, we can predict from our results that the L1 activity might be very low or even lost in the sloth and hyrax genomes (fig. 5 and table 2).

Our work also reveals that mammalian L1 and LINE-1-like elements are not the only ones capable of mobilizing snRNA sequences. We have found members of three different LINE clades that were able to generate U6 pseudogenes (i.e., L1, L2, and RTE). However, unlike L1, for which U6 pseudogenes may have different structures, LINEs from other clades (L2, RTE) form only chimeras through the template switching mechanism. Interestingly, these clades represent LINEs with “stringent” protein/RNA interaction models (Okada et al. 1997; Kajikawa and Okada 2002; Hayashi et al. 2014). Here, we show that template switching has been continuously active at least in genomes from birds to mammals. This reinforces the finding that template switching is an ancient property of LINE elements. Indeed, template switching has been proposed to explain the generation of a new type of repeat in the rice blast fungus *Magnaporthe grisea* (MINE element) (Fudal et al. 2005; Gogvadze et al. 2007). In this fungus genome, as in mouse and human, triple chimera were also observed (Gogvadze et al. 2005, 2007). Such peculiar tripartite structures can be explained either by double template switching during a unique insertion event or by two independent retrotransposition events, the second occurring inside the 3′-extremity of the first insertion. Altogether, it reinforces the notion that template switching mechanism, associated with many LINE clades, may have played a major role in SINE–LINE coevolution. Indeed, early studies have proposed that this mechanism is responsible for the transfer of the 3′-extremity of stringent LINEs to tRNA-derived sequences leading to the emergence of new SINEs families (Gilbert and Labuda 1999; Ohshima and Okada 2005).

## Materials and Methods

### In Silico Analysis for the Human Genome

Sequences of the human snRNA genes were obtained at GenBank (http://www.ncbi.nlm.nih.gov/genbank/, last accessed March 20, 2015) using the following accession numbers: U1 (J00318), U2 (K02227), U4 (M15957), U5 (X04215), U6 (M14486), U11 (X58716), U12 (J04119), U4atac (U62822), and U6atac (U62823). The obtained sequences were used to perform BLAST search (using default parameters; Altschul et al. 1997) on Ensembl release 73 (http://www.ensembl.org/index.html, last accessed March 20, 2015). We limited our analysis to sequences having at least 90% identity with the reference gene and retrieved 3,512 sequences. For U2 and U6, due to the large number of hits, we further limited our analysis to sequences with at least 97.5% identity. For all snRNAs, we next restricted our analysis to sequences larger than 25 nucleotides (450 sequences). A closer look at the shortest sequences, generally truncated at their 5′- and 3′-ends, revealed that they largely corresponded to longer sequences carrying one or two mismatches in the first or last 10–15 nucleotides (segments of the sequence omitted by the BLAST program). We then excluded these shortest sequences from the analysis as their combined segments correspond to longer sequences with low levels of identity (<90% or 97.5% to the reference sequence). Hits with sequences not assigned to a specific locus were also excluded from the analysis. For example, the U2 snRNA gene is not included in the analysis since no data were available for its precise chromosomal location (table 1). Also, snRNA sequences that are part of large genomic duplications (meaning that the 5′- and 3′-flanking sequences of the snRNA are also repeated) were considered only once in the analysis. The sequence with the highest identity was saved and the duplicated copies were excluded. For example, 24 copies of U1 snRNA are present in a cluster on chromosome 1, most likely due to genomic duplication. After applying all these restrictive parameters, we retrieved 256 snRNA sequences. These sequences appear to be randomly distributed throughout the genome.

Each sequence identified was used independently for a BLAT search (http://genome.ucsc.edu/cgi-bin/hgBlat, last accessed March 20, 2015) to identify repeat sequences present in the flanking region (using RepeatMasker tool on the genome browser). Each TSD was annotated by hand, and was defined as the longest identical segment at each extremity of the processed pseudogene. Thus, for a number of sequences, several nucleotides can be considered as part of the TSD but may also correspond to the retrotransposed sequence (fig. 3). For 3′-truncated snRNA copies, consensus cleavage sites were obtained using WebLogo (Crooks et al. 2004).

Detailed information for each processed snRNA pseudogene analyzed in this study can be provided upon request.

### Analyzed Genomes

All the 48 analyzed genomes are indicated in table 2 and are accessible on the Ensembl web site: http://www.ensembl.org/info/about/species.html (last accessed March 20, 2015). In our analysis, we used the *Homo sapiens* U6 snRNA reference sequence mentioned above (GenBank accession number M14486), as it is conserved in all mammals (with 100% identity). Although no annotated U6 snRNA gene was available for the lizard and birds genome, the use of human U6 snRNA sequence in an initial BLAST search allowed us to confirm the extended conservation of U6 snRNA sequence to the lizard and birds genomes. We used the xenopus U6 snRNA reference sequence (GenBank accession number NR_033272) to screen the xenopus genome.

### Bioinformatic Analysis

The program, named ProRNAScan, was created to analyze the snRNA pseudogenes from the 48 genomes mentioned above, and is available at http://endorphine.igh.cnrs.fr (last accessed March 20, 2015). Other genomes, available from the Ensembl web site, can be added upon request. The web interface was made interactive and efficient by using Ajax and JQuery. The backend service was developed using Perl on an Apache web server. The pipeline is set by default with the selective parameters established for the human U6 snRNA analysis (i.e.*,* 97.5% identity to the referring sequence and at least 26 nucleotides in length, 10 for the *e* value and 10 for the size of the TSD). The end user can modify these parameters.

For detection of small noncoding retrotransposed sequences, the program initially performs an homology search using BLAST (with the default parameters), generating a first set of potential hits. For each hit, 100 nucleotides upstream and downstream of the sequence are collected.

In order to define the structure of the inserted sequence and to classify them into four groups (“Alone,” “Repeat,” “PolyA,” and “3′-truncated”), the program performs successively three steps of analysis. In the first step, the program identifies the potential TSD signature with a local alignment using EMBOSS wordmatch, which identifies exact matches between two sequences (Rice et al. 2000). The first and last ten nucleotides of the hit itself are also included in the TSD search to allow for some boundary imprecision. By default, TSDs were required to be at least ten nucleotides long. If no match is found, the downstream sequence is extended to 6 kb in order to find more distant TSD, and the program runs successively EMBOSS wordmatch and wordfinder, which allows mismatches in the alignment. Next, if TSD is found, the program tests for the presence of repeat sequences by performing BLAST (with default parameters) against RepBase Update, a library of mobile elements (Jurka et al. 2005). When found, the repeat family is specified in the result. When not found, we have to manually look for the origin of the sequence (see below). Finally, a search of poly(A) sequences is performed and is positive if more than six adenosines are present in a sliding window of ten nucleotides. After each step, if the result is positive, the downstream sequence is rebuilt and the boundary of the signature is recorded. Next to the four defined groups, we added two other categories: “To Check” and “Gaps.” The “To Check” category includes sequences that are 5′-truncated and sequences that are 3′-truncated without TSD. The “Gaps” category includes sequences where unsequenced gaps (succession of Ns in the assembled genome) were found within the boundaries. All copies landing in one of these two groups were curated manually and either redistributed to the appropriate group or excluded from the analysis if they did not fit our selective criteria. An illustration is displayed in supplementary figure S1, Supplementary Material online, including information about output options.

When a “repeat” sequence associated with an snRNA copy is not found in RepBase, such as processed pseudogenes or nonannotated repeat sequence, we manually take the sequence and perform a BLAT search (http://genome.ucsc.edu/cgi-bin/hgBlat, last accessed March 20, 2015). This search allows us to identify the origin of the “repeat” sequence, for example a precursor gene (located elsewhere in the genome and containing introns) or a possible not yet identified repeat.

It is important to note that not all genomes are at the same stage of assembly and their repeat content has not been equally defined. The difference in quality may have introduced biases in our analysis. One bias could result in a lowered estimate of the number of sequences for a subset of genomes. A second bias could be the presence of gaps (undefined nucleotides) near the pseudogene that would prevent the characterization of the insertion. The supplementary table S3, Supplementary Material online, regroups the available information regarding each analyzed genome: Size, repeat content (if known), sequencing coverage, and scaffold average size for the latest genome assembly version.

ProRNAScan is not flawless, and several errors in the attribution of a sequence to a defined group (Alone, repeat, poly(A) and 3′-trunc) have been observed among all analyzed genomes. We manually estimated the error frequency between less than 5% and 15%, depending on the genome. Nevertheless, we also noticed that errors compensate themselves and the final distribution after correction was always similar (data not shown).

### Analysis of Junction Homology

The expected distribution of junction homology at the 3′-junction of 3′-truncated snRNA copies was calculated according to Roth et al. (1985). The probability to observe a sequence of *n* homologies is computed as *P*(*n*) = (*n* + 1) · *p^n^* · (1 − *p*)^2^, where *p* denotes the probability of random homology of a single nucleotide. Here, *p* was set to 0.25 assuming an unbiased base composition of the target sequences. To compare with the number of observed distribution of junction homology, we multiplied *P*(*n*) by the total number of events analyzed.

### Statistic: Fisher’s Exact Test

To evaluate the variability of the observed proportion of each structural group of U6 snRNAs, all the genomes were compared against each other using a Fisher’s exact test. This test is equivalent to the chi-square test, but it is better suited for contingency tables that have columns (or lines) with sum equal to zero. The contingency table of each side-by-side comparison is made from two variables: 1) The structural group of the U6 snRNA sequences (Alone, Repeat, Poly(A), 3′-trunc) and 2) two genomes (among the 48 analyzed). Then, a table has been produced with the genomes in abscissa and ordinate, where each cell contains the *P* value of the Fisher’s exact test that has been calculate for two species (supplementary table S2, Supplementary Material online).

## Supplementary Material

Supplementary tables S1–S3 and figure S1 are available at *Molecular Biology and Evolution* online (http://www.mbe.oxfordjournals.org/).

Supplementary Data
