# Supplementary material for: U6 snRNA Pseudogenes: Markers of Retrotransposition Dynamics in Mammals
Source: Mol Biol Evol. 2015 Mar 11;32(7):1815–32. doi: 10.1093/molbev/msv062 (PMC4476161; doi:10.1093/molbev/msv062)
Supplement: Supplementary Data [file supp_32_7_1815__index.html]

U6 snRNA Pseudogenes: Markers of Retrotransposition Dynamics in Mammals — U6 snRNA Pseudogenes: Markers of Retrotransposition Dynamics in Mammals — Supplementary Data 

# U6 snRNA Pseudogenes: Markers of Retrotransposition Dynamics in Mammals

## Supplementary Data

files

**Files in this Data Supplement:**

- Supplementary Data - pdf file
- Supplementary Data - xlsx file
