# Supplementary material for: U6 snRNA Pseudogenes: Markers of Retrotransposition Dynamics in Mammals
Source: Mol Biol Evol. 2015 Mar 11;32(7):1815–32. doi: 10.1093/molbev/msv062 (PMC4476161; doi:10.1093/molbev/msv062)
Supplement: Supplementary Data [file supp_msv062_Supplemental_Data_Doucet_et_al.pdf]

Supplemental Data

Supplemental Table 1: snRNA sequences associated with repeats. Details of the 30 sequences that have been found associated with retrotransposons or processed pseudogenes. [a] enumeration and the type of the snRNA genomic copy. [b] nucleotide length of the associated repeat. [c] size of the TSD ("del" means a short deletion, "na" stands for not applicable). [d] first strand cleavage site based on the snRNA transcriptional orientation. [e] 3' truncation position of the snRNA sequence. [f] number of nucleotide homologies at the junction between the snRNA and the flanking sequence for twin priming-like insertions. A* indicates the presence of an extra A nucleotide between the U6atac and the *Alu* sequence. [g] specifies if the chimera with a repeat sequence originates from a template switching mechanism or a twin priming-like mechanism (twin). In parenthesis, for the latter, the cleavage site is provided in the repeat transcriptional orientation. For sequence number 5 and 16, details of the insertion site were built by comparing with other primate genomes (indicated in parenthesis).

Supplemental Table 2: Fisher's exact test. This table displays the results of the Fisher's exact test conducted between species used in this study. The species names are indicated both in abscissa and ordinate (row and column headers). Each cell contains the *p*-value of the Fisher's exact test that has been calculated between the two species indicated in the column and row headers. The test is performed using two variables: (i) the structural group of the U6 snRNA sequences (Alone, Repeat, Poly(A), 3'trunc) and (ii) 2 genomes (among the 48 analysed). To make this table more readable, subgroups of cells corresponding to the same phylogenetic order have been colored (chiroptera in brown, carnivora in blue, artiodactyla in green, primates in red, rodentia in orange). Inside these sub-tables, cells with a *p*-value above

0.01, suggesting similar distribution of each groups of processed pseudogenes between the two genomes, are filled with lighter colors.

Supplemental Table 3: Repeat content of genomes. [a] and [b], scientific and common names of the analysed genomes, respectively. [c] and [d], genome size and coverage, respectively. [e] to [i], estimated proportion of the genome for each category of repeated sequences: LINEs, SINEs, LTR retrotransposons (LTR retro.), DNA transposons (DNA trans.) and unclassified (others), respectively. [j] total percentage of the genome covered by repeated sequences. [k] N50 scaffold, * a longer N50 usually indicates a more complete genome assembly. [l] total number of hits, found by BLAST search, with identity to U6 sequence. [m] number of hits that reach our selective criteria. [n] number of hits with gaps (non-assigned nucleotides). Details of the data provided from [a] to [l] are accessible on the *Ensembl* genome list browser (http://www.ensembl.org/info/about/species.html). nd is used for no data.

Supplemental Figure 1: Illustration of the bioinformatic analysis using ProRNAScan. Panel A is a picture of the web interface of ProRNAScan. U6 snRNA sequence can be uploaded as an example but any small non-coding gene could be used for analysis. Genomes can be chosen from the list provided from *Ensembl*. Selective parameters are set by default but allow setting the minimum identity to the referring sequence, the minimal size of TSD and the minimal size of sequence homology. Panel B is a picture of the results obtained on the web interface. Depending on the results, a maximum of 9 files compiling the results can be downloaded. "Download result as fasta file" is a text file with all the retrieved snRNA sequences, their associated segment when applicable (repeat or poly(A)) and an extra 100 bp genomic DNA sequence upstream and downstream. "Download result as fasta file (Only To Check)" is a text file with all the sequences that need to be checked before being associated to

a particular group. “Download result as fasta file (Only Alone)”, “Download result as fasta file (Only Repeat)”, “Download result as fasta file (Only PolyA)”, “Download result as fasta file (Only 3' truncated)”, are text files with all sequences of each group separately. “Download result as tab file” is a text file with a description of all retrieved sequences, providing notably the genomic position of the identified sequence and the group it belongs to, the poly(A) position, sequence and size, the TSD position, sequence and size. “Download result as tab file (Only TSD)” is similar as the last file but contains only the results for sequences with TSD. Finally, “Download result as Excel file” is an excel file that summarizes all the results.

Additional references for Supplemental Table 2

Alfoldi J, Di Palma F, Grabherr M, Williams C, Kong L, Mauceli E, Russell P, Lowe CB, Glor RE, Jaffe JD et al. 2011. The genome of the green anole lizard and a comparative analysis with birds and mammals. *Nature* **477**(7366): 587-591.

Consortium CGS. 2004. Sequence and comparative analysis of the chicken genome provide unique perspectives on vertebrate evolution. *Nature* **432**(7018): 695-716.

Dalloul RA, Long JA, Zimin AV, Aslam L, Beal K, Blomberg Le A, Bouffard P, Burt DW, Crasta O, Crooijmans RP et al. 2010. Multi-platform next-generation sequencing of the domestic turkey (Meleagris gallopavo): genome assembly and analysis. *PLoS Biol* **8**(9).

Elsik CG Tellam RL Worley KC Gibbs RA Muzny DM Weinstock GM Adelson DL Eichler EE Elnitski L Guigo R et al. 2009. The genome sequence of taurine cattle: a window to ruminant biology and evolution. *Science* **324**(5926): 522-528.

Fang X, Mou Y, Huang Z, Li Y, Han L, Zhang Y, Feng Y, Chen Y, Jiang X, Zhao W et al. 2012. The sequence and analysis of a Chinese pig genome. *Gigascience* **1**(1): 16.

Gibbs RA Weinstock GM Metzker ML Muzny DM Sodergren EJ Scherer S Scott G Steffen D Worley KC Burch PE et al. 2004. Genome sequence of the Brown Norway rat yields insights into mammalian evolution. *Nature* **428**(6982): 493-521.

Hellsten U, Harland RM, Gilchrist MJ, Hendrix D, Jurka J, Kapitonov V, Ovcharenko I, Putnam NH, Shu S, Taher L et al. 2010. The genome of the Western clawed frog Xenopus tropicalis. *Science* **328**(5978): 633-636.

Lander ES Linton LM Birren B Nusbaum C Zody MC Baldwin J Devon K Dewar K Doyle M FitzHugh W et al. 2001. Initial sequencing and analysis of the human genome. *Nature* **409**(6822): 860-921.

Lindblad-Toh K Wade CM Mikkelsen TS Karlsson EK Jaffe DB Kamal M Clamp M Chang JL Kulbokas EJ, 3rd Zody MC et al. 2005. Genome sequence, comparative analysis and haplotype structure of the domestic dog. *Nature* **438**(7069): 803-819.

Mikkelsen TS, Wakefield MJ, Aken B, Amemiya CT, Chang JL, Duke S, Garber M, Gentles AJ, Goodstadt L, Heger A et al. 2007. Genome of the marsupial Monodelphis domestica reveals innovation in non-coding sequences. *Nature* **447**(7141): 167-177.

Petersen JL, Mickelson JR, Rendahl AK, Valberg SJ, Andersson LS, Axelsson J, Bailey E, Bannasch D, Binns MM, Borges AS et al. 2013. Genome-wide analysis reveals selection for important traits in domestic horse breeds. *PLoS Genet* **9**(1): e1003211.

Renfree MB Papenfuss AT Deakin JE Lindsay J Heider T Belov K Rens W Waters PD Pharo EA Shaw G et al. 2011. Genome sequence of an Australian kangaroo, Macropus eugenii, provides insight into the evolution of mammalian reproduction and development. *Genome Biol* **12**(8): R81.

Warren WC, Clayton DF, Ellegren H, Arnold AP, Hillier LW, Kunstner A, Searle S, White S, Vilella AJ, Fairley S et al. 2010. The genome of a songbird. *Nature* **464**(7289): 757-762.

Waterston RH Lindblad-Toh K Birney E Rogers J Abril JF Agarwal P Agarwala R Ainscough R Alexandersson M An P et al. 2002. Initial sequencing and comparative analysis of the mouse genome. *Nature* **420**(6915): 520-562.

Whittington CM, Papenfuss AT, Locke DP, Mardis ER, Wilson RK, Abubucker S, Mitreva M, Wong ES, Hsu AL, Kuchel PW et al. 2010. Novel venom gene discovery in the platypus. *Genome Biol* **11**(9): R95.

	type [a]	length [b]	TSD [c]	cleavage [d]	3'truncation position [e]	junction [f]	remarks [g]
1	U1_3'trunc-L1	623	17	CTTTC-TG	40	3	L1-twin (TTTCT-AT)
2	U2_3'trunc-L1	1457	14	TTCAA-GA	55	2	L1-twin (CTTTT-AA)
3	U5_3'trunc-L1	394	20	AGGTG-GA	36	1	L1-twin (TTTTC-AG)
4	U5_3'trunc-SVA	479	15	GTTAG-AA	48	4	SVA-twin (CTTTC-AA)
5	U5_3'trunc-L1	700	16	GTTTT-CA	83	na	U5-L1 (TSD from rhesus genome)
6	U5_3'trunc-L1	3712	15	GAGGA-GC	43	1	L1-twin (TTTTA-AT)
7	U5_3'trunc-L1	38	17	AGTTA-CA	46	5	L1-twin (TTTCT-AA)
8	U5_3'trunc-L1	1084	8	AGTTT-CT	78	na	U5-L1
9	U5_3'trunc-un.	29	15	TTTTT-AA	98	na	U5-sequence unknown
10	U6_3'trunc-L1	121	7	AAAGC-TC	68	2	L1-twin (TTTTC-AA)
11	U6-L1	1365	19	TTTTT-AA	full length	na	U6-L1
12	U6-L1	760	15	TTCTT-GA	full length	na	U6-L1
13	U6-L1	119	16	TCTTT-GC	full length	na	U6-L1
14	U6-L1	3464	14	TCTTT-GA	full length	na	U6-L1
15	U6-L1	438	13	TTTTT-TT	full length	na	U6-L1
16	U6-L1	4416	del	TGTAA-AT	full length	na	U6-L1 (del. from chimpanzee genome)
17	U6-L1	4885	15	TTCTT-AA	full length	na	U6-L1
18	U6-L1	1173	17	ATTTT-AA	full length	na	U6-L1
19	U6-L1	595	17	TTTTA-AT	full length	na	U6-L1
20	U6-L1	1343	11	TTCTT-AA	full length	na	U6-L1
21	U6-L1	1374	13	TCTTT-AA	full length	na	U6-L1
22	U6-L1	2103	16	TTTTT-AA	full length	na	U6-L1
23	U6-pseudogene	1314	15	TTTTT-AA	full length	na	U6-pseudo (ACAA2)
24	U6atac-L1	383	14	GTTTT-AT	full length	na	U6atac-L1
25	U6atac_3'trunc-L1	268	13	GATTT-CA	80	na	U6atac-L1
26	U6atac-Alu	294	na	na	25-125	A*	U6atac-Alu
27	U6atac-L1	1262	13	ATCTT-AA	full length	na	U6atac-L1
28	U6atac-L1	1237	14	ATTTT-AA	full length	na	U6atac-L1
29	U6atac-L1	195	12	TTCTT-AA	full length	na	U6atac-L1
30	U6atac-Alu	311	14	TCTTT-AT	full length	na	U6atac-Alu

Supplemental Table 1 : Doucet *et al.*

[a]	Common name [b]	size [c]	coverage [d]	LINE [e]	SINE [f]	LTR retro [g]	DNA trans [h]	others [i]	total [j]	scaffold N50*[k]	Total U6 hits [l]	hits selected [m]	hits with gap [n]	
Xenopus tropicalis	Xenopus	1510	7X	3,1	3,8	1,75	25	0,5	34,15	124,127,367	48	18	0	Hellsten et al. 2010
Gallus gallus	Chicken	1046	12X	6,4	0,1	1,3	0,8		8,6	12,877,381	41	4	0	Consortium 2004
Meleagris gallopavo	Turkey	1130	35X	4,81		0,51	0,65	0,98	6,95	3,801,642	32	4	1	Dalloul et al. 2010
Taeniopygia guttata	Zebra finch	1230	5,5X	3,3	0,06	3,92	0,01		7,29	8,236,790	10	3	1	Warren et al. 2010
Anolis carolinensis	Lizard	1800	7X	12	5	2,9	8,6		28,5	4,033,265	192	52	0	Alfoldi et al. 2011
Ornithorhynchus anatinus	Platypus	1995	6X	21,04	22,43	0,15	0,56	0,45	44,63	958,97	412	70	9	Whittington et al. 2010
Sarcophilus harrisii	Tasmanian devil	3170	85X	nd	nd	nd	nd		nd	1,847,106	297	16	4	
Macropus eugenii	Wallabi	3075	2X	28,6	11,7	3,9	2,9		47,1	36,602	252	7	3	Renfree et al. 2011
Monodelphis domestica	Opossum	3600	7X	29,2	10,4	10,6	1,7		51,9	59,809,810	721	16	0	Mikkelsen et al. 2007
Choloepus hoffmanni	Sloth	2460	2X	nd	nd	nd	nd		nd	366,442	253	15	2	
Dasypus novemcinctus	Armadillo	3630	6X	nd	nd	nd	nd		nd	1,687,935	604	218	34	
Echinops telfairi	Tenrec	2950	78X	nd	nd	nd	nd		nd	45,764,842	263	72	4	
Loxodonta africana	Elephant	3200	7X	nd	nd	nd	nd		nd	46,401,353	779	20	2	
Procavia capensis	Hyrax	3600	2X	nd	nd	nd	nd		nd	905,827	257	9	2	
Erinaceus europaeus	Hedgehog	2710	79X	nd	nd	nd	nd		nd	3,264,618	258	93	11	
Sorex araneus	Shrew	2420	120X	nd	nd	nd	nd		nd	22,794,405	274	111	23	
Myotis lucifugus	Microbat	2030	7X	nd	nd	nd	nd		nd	4,293,315	918	126	2	
Pteropus vampyrus	Megabat	2200	3X	nd	nd	nd	nd		nd	5,954,017	296	9	0	
Equus caballus	Horse	2470	7X	19,56	7,46	6,27	3,15	11,27	47,71	46,749,900	416	27	1	Petersen et al. 2013
Felis catus	Cat	2450	6X	14,26	11,2	4,44	2,19		32,09	4,658,941	1918	322	21	Pontius et al. 2007
Canis familiaris	Dog	2400	7X	18,74	10,57	3,68	1,98		34,97	45,876,610	2849	454	1	Lindblad-Toh et al. 2005
Ailuropoda melanoleuca	Panda	2400	2X	nd	nd	nd	nd		nd	1,281,781	469	32	4	
Mustela putorius furo	Ferret	2400	162	nd	nd	nd	nd		nd	9,335,154	2519	587	12	
Tursiops truncatus	Dolphin	2550	2X	nd	nd	nd	nd		nd	116,287	294	43	8	
Sus scrofa	Pig	2800	24X	18,1	13,6	4,5	2,2		38,4	576,008	905	54	2	Fang et al. 2012
Bos taurus	Cow	2670	9X	23,3	17,67	3,62	1,96		46,55	6,380,747	1099	148	4	Elsik et al. 2009
Ovis aries	sheep	2600	142X	nd	nd	nd	nd		nd	100,079,507	1085	171	10	
Vicugna pacos	Alpaca	2170	22X	nd	nd	nd	nd		nd	7,263,804	310	48	3	
Microcebus murinus	Mouse lemur	2900	2X	nd	nd	nd	nd		nd	107,020	287	68	5	
Otolemur garnettii	Bushbaby	2500	173X	nd	nd	nd	nd		nd	13,852,661	2457	162	22	
Tarsius syrichta	Tarsier	3200	2X	nd	nd	nd	nd		nd	10,450	256	21	3	
Callithrix jacchus	Marmoset	2750	6X	nd	nd	nd	nd		nd	5,167,444	1963	179	22	
Macaca mulatta	Macaque	3200	4,6X	nd	nd	nd	nd		nd	5,874,613	1344	73	6	
Papio anubis	Olive baboon	2950	85X	nd	nd	nd	nd		nd	528,927	1370	65	5	
Chlorocebus sabaeus	Vervet-AGM	2800	95X	nd	nd	nd	nd		nd	81,825,804	1385	45	0	
Pongo abelii	Orangutan	3400	12X	nd	nd	nd	nd		nd	747,460	1422	39	7	
Gorilla gorilla	Gorilla	3050	35X	nd	nd	nd	nd		nd	913,458	1401	47	12	
Pan troglodytes	Chimpanzee	3300	6X	nd	nd	nd	nd		nd	8,925,874	1466	50	5	
Homo sapiens	Human	3209		20,42	13,29	8,29	2,84		44,84	67,794,873	1515	55	0	Lander et al. 2001
Nomascus leucogenys	Gibbon	2950	15X	nd	nd	nd	nd		nd	52,956,880	1455	71	5	
Tupaia belangeri	Tree shrew	3660	low	nd	nd	nd	nd		nd	88,860	279	80	12	
Ochotona princeps	Pika	2220	low	nd	nd	nd	nd		nd	26,863,993	306	136	13	
Oryctolagus cuniculus	Rabbit	2730	7X	nd	nd	nd	nd		nd	35,972,871	1140	181	3	
Cavia porcellus	Guinea Pig	2700	7X	nd	nd	nd	nd		nd	27,942,054	1416	73	0	
Dipodomys ordii	Kangaroo rat	2200	2X	nd	nd	nd	nd		nd	11,931,245	276	47	11	
Mus musculus	Mouse	2800	67X	19,21	8,22	9,87	0,88		38,18	52,589,046	904	159	0	Waterston et al. 2002
Rattus norvegicus	Rat	2900	6X	23,11	7,05	9,04	0,81		40,01	2,178,346	1010	295	34	Gibbs et al. 2004
Ictidomys tridecemlineatu	Squirrel	2480	495X	nd	nd	nd	nd		nd	8,192,786	829	44	9	

Supplemental Table 3: Doucet *et al.*

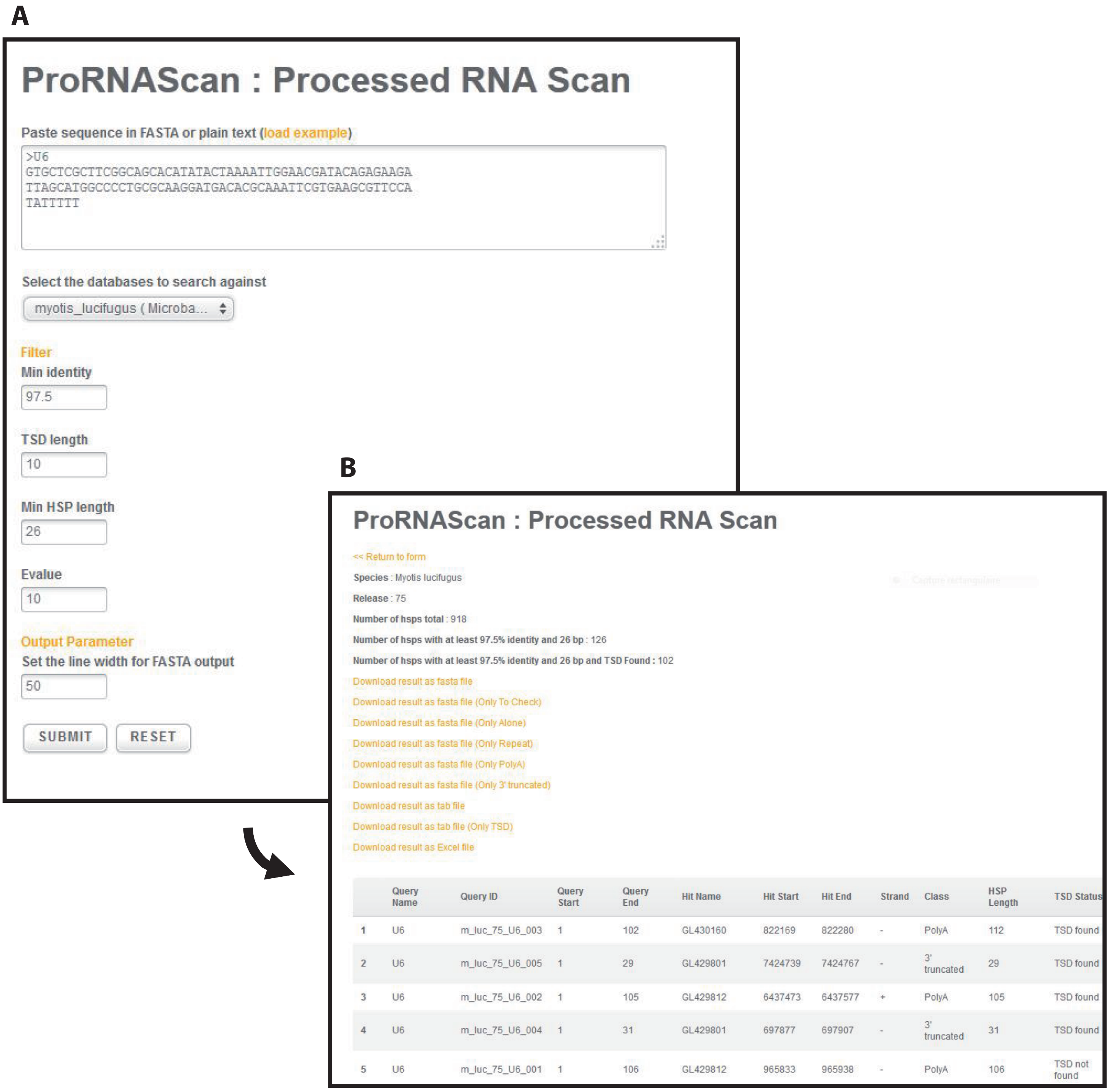

Supplemental Figure 1 : Doucet *et al.*
